# Monosodium Urate Crystal Depletion and Bone Erosion Response in Kidney Transplant Recipients With Uncontrolled Gout Treated With Pegloticase: PROTECT Serial Dual-Energy Computed Tomography Findings

**DOI:** 10.1097/TXD.0000000000001803

**Published:** 2025-05-12

**Authors:** Nicola Dalbeth, Abdul Abdellatif, John K. Botson, Kenneth G. Saag, Ada Kumar, Lissa Padnick-Silver, Zana Vranic, Brad A. Marder, Fabio Becce

**Affiliations:** 1 Department of Medicine, University of Auckland, Auckland, New Zealand.; 2 Division of Nephrology, Kidney Hypertension Transplant Clinic, Baylor College of Medicine, Houston, TX.; 3 Orthopedic Physicians Alaska, Anchorage, AK.; 4 Division of Rheumatology, Department of Medicine, University of Alabama at Birmingham, Birmingham, AL.; 5 Amgen Inc, Thousand Oaks, CA.; 6 Department of Diagnostic and Interventional Radiology, Lausanne University Hospital, University of Lausanne, Lausanne, Switzerland.

## Abstract

**Background.:**

Hyperuricemia and gout are associated with poor outcomes in kidney transplant (KT) recipients, including graft failure. The PROspective sTudy of pEglotiCase in Transplant patients (PROTECT) trial showed high urate-lowering efficacy of pegloticase in immunosuppressed KT recipients with uncontrolled gout. Here, we report serial dual-energy computed tomography (DECT) findings in PROTECT participants.

**Methods.:**

KT recipients with uncontrolled gout (serum urate [SU] ≥7 mg/dL, refractory to/intolerant of oral urate-lowering therapy, and symptoms [≥2 flares per year, tophi, and/or gouty arthritis]) and serial DECT imaging were included. Patients were required to have an estimated glomerular filtration rate ≥15 mL/min/1.73 m^2^ >1 y posttransplant. All patients received pegloticase for ≤24 wk (8 mg infusion every 2 wk) and underwent imaging (screening, week 14, week 24). DECT images were acquired with standard protocols and postprocessed for monosodium urate (MSU) volume (V_MSU_) using default settings. Regions (bilateral hands/wrists, feet/ankles, knees) with paired screening/week 24 images and screening V_MSU_ ≥0.5 cm^3^ (minimized DECT-artifact influence) were included.

**Results.:**

Eight patients underwent DECT imaging (all men; age: 52.3 ± 11.2 y, time since KT: 18.7 ± 6.9 y, estimated glomerular filtration rate: 45.6 ± 12.4 mL/min/1.73 m^2^, SU: 10.4 ± 2.1 mg/dL). Six patients (75%) completed the study and received 24 wk of pegloticase therapy, and 2 prematurely discontinued because of COVID-exposure concerns. Of the 6 patients, 4 met imaging inclusion criteria and were included in the analysis. All 4 patients had sustained SU-lowering during month 6 with marked V_MSU_ reduction at week 24 (mean change in V_MSU_: –98.9%±1.7% [5 imaging regions]). Numerous bone erosions were present in all patients with MSU-adjacent, unknown mineral deposit-adjacent, and deposit-independent erosions. Imaging suggested osteopenia/osteomalacia in 5 patients (83%). After pegloticase treatment, MSU-adjacent erosions decreased in size in a single patient with no DECT evidence of osteopenia/osteomalacia.

**Conclusions.:**

Consistent with prior studies in nontransplant populations, marked depletion of deposited MSU occurred in KT recipients with uncontrolled gout after pegloticase therapy. However, unlike transplant-naive patients, subsequent bone erosion remodeling was not widely observed in urate-adjacent erosions, perhaps due to overall poor bone health in this patient population.

**Clinical Trial Registration.:**

ClinicalTrials.gov: NCT04087720.

Because urate is primarily eliminated via renal excretion, patients with compromised kidney function are at an increased risk for both hyperuricemia^1^ and gout.^2^ Kidney transplant (KT) recipients are at even greater risk, with the prevalence of gout in KT recipients reported at 12 times greater than in the general nontransplanted US population.^3^ Unfortunately, oral urate-lowering therapies are often limited in KT recipients due to impaired renal function and potential drug interactions.^4^ Therefore, serum urate (SU) levels often remain high (defined as ≥7 mg/dL in men and ≥6.0 mg/dL in women) in KT recipients, resulting in subsequent monosodium urate (MSU) crystal deposition that causes corresponding symptoms in joints, bones, and soft tissues.^5,6^

Prior studies using dual-energy computed tomography (DECT) have confirmed a physical relationship between MSU crystal deposits and adjacent bone erosions.^7^ MSU crystal deposits adjacent to bones can lead to erosions at the bone-tophus interface^7^ from the resulting osteoblast/osteoclast activity imbalance.^8^ Of importance, growing evidence using DECT imaging suggests that gout-related bone erosions begin to remodel after urate depletion in pegloticase-treated non-KT patients.^6,9^ Given that KT recipients are at an increased risk for developing chronic kidney disease-mineral and bone disorder (CKD-MBD), including renal osteodystrophy, osteoporosis, osteonecrosis, and fracture^10^ and that patients with comorbid gout and CKD may be more vulnerable to gout-related bone damage,^11^ better understanding of the bone-related consequences of gout in KT recipients is of importance.

The PROspective sTudy of pEglotiCase in Transplant patients (PROTECT) trial demonstrated urate-lowering efficacy of pegloticase in most KT recipients on a stable immunosuppression regimen with no safety concerns (89% had sustained SU <6 mg/dL during treatment month 6).^12^ Subsequent progressive improvements with urate-lowering were also observed in both clinical and quality-of-life measures.^13^ A subset of PROTECT participants underwent serial DECT imaging during pegloticase treatment, but findings have not yet been reported. Here, we describe MSU deposition volume (V_MSU_) changes with pegloticase treatment and also report on bone-related observations.

## PATIENTS AND METHODS

The phase 4, multisite, open-label PROTECT clinical trial (NCT04087720) was conducted in accordance with the principles of the Declaration of Helsinki. An institutional review board or ethics committee at each study site reviewed and approved the study protocol. Written informed consent for study participation was obtained from all patients before performing any study-related examinations or procedures. Study methods have been previously described in full,^12^ but are additionally described here in brief.

### Patients

This clinical trial included adult KT recipients with uncontrolled gout, defined as SU ≥7 mg/dL, oral urate-lowering therapy inefficacy or intolerability, and ≥1 ongoing signs per symptom of gout: ≥1 tophus, ≥2 gout flares in the prior year, and/or chronic gouty arthritis. All patients were at least 1 y posttransplant and on a stable immunosuppressant regimen for at least 3 mo before study screening. Patients were required to have a functional graft (estimated glomerular filtration rate ≥15 mL/min/1.73 m^2^) and be able to tolerate low-dose prednisone (<10 mg/d) as gout flare prophylaxis (initiated ≥1 wk before first pegloticase infusion). Key exclusion criteria included unresolved severe infection within 2 wk before day 1, chronic or active hepatitis B infection, history of hepatitis C virus RNA positivity (unless treated and undetectable), history of HIV positivity, glucose-6-phosphate deficiency, congestive heart failure, uncontrolled arrhythmia, or uncontrolled hypertension (>160/100 mm Hg) at the end of the screening period.

### Study Design

The full study design has been previously described.^12^ Briefly, the PROTECT open-label trial examined the efficacy and safety of pegloticase in adult KT recipients with uncontrolled gout. The study’s primary endpoint was the proportion of patients with SU-lowering during month 6 of treatment (SU <6 mg/dL for ≥80% of weeks 20–24). All patients who continued to meet study criteria through the screening period (≤35 d) entered a 24-wk pegloticase treatment period (8 mg infusion every 2 wk; 12 infusions). All patients received low-dose prednisone as flare prophylaxis for ≥1 wk before the first infusion and standard preinfusion prophylaxis before each pegloticase dose. Patients completed a safety visit via phone or email 30 d after the last pegloticase infusion and a full clinical assessment 3 mo after the last pegloticase infusion.

### DECT Imaging

DECT images were acquired using standard protocols at screening (baseline), week 14, and week 24. MSU crystal deposition volume (V_MSU_) was automatically measured using default postprocessing settings. Patients with paired screening and week 24 images were included in DECT analyses. Imaged regions with a screening V_MSU_ <0.5 cm^3^ were excluded to minimize contributions of possible DECT artifacts.^14^ Included imaged regions were assessed for radiological bone findings. Erosions were assessed by a centralized reader based on the OMERACT RAMRIS semiquantitative scoring system. Erosive damage at each location (hands and feet) was scored from 0 to 10, with each score representing a 10% incremental loss of bone. The total erosion score represented is the additive percent. Although bone analyses beyond erosion changes were not planned, pertinent findings are reported for completeness.

### Statistical Methods

Statistical methods for the study, including the primary endpoint, safety analysis, pharmacokinetics, and pegloticase immunogenicity evaluations, have been fully described elsewhere.^12^ Results of the current exploratory DECT analyses were examined using descriptive statistics and are presented as mean (±SD), mean (range), or median (range) for continuous parameters and n (%) for categorical parameters. Unless otherwise specified, values at screening were used as the baseline.

## RESULTS

### Patients

A total of 8 patients underwent serial DECT imaging. The mean patient age was 52.3 ± 11.2 y, all patients were men, and the mean body mass index was 32.3 ± 8.7 kg/m^2^. The mean time since KT was 18.7 ± 6.9 y (range, 8.5–27.6) before study enrollment, and the mean estimated glomerular filtration rate at screening was 45.6 ± 12.4 mL/min/1.73 m^2^ (range, 25.0–63.8). Patients had an 11.9 ± 17.0-y history of gout (based on first gout diagnosis), and the screening SU level averaged 10.4 ± 2.1 mg/dL (Table 1). Of the 8 patients with DECT imaging, 6 (75%) completed the study (24 wk of treatment, 12 pegloticase infusions), and 2 discontinued the study prematurely due to COVID concerns (2 and 4 infusions received, respectively) and thus did not have paired images for week 24 and screening. Of the 6 patients who completed 24 wk of treatment, 2 did not meet imaging inclusion criteria (screening V_MSU_ <0.5 cm^3^ in the 1 imaged region of each patient). Therefore, 4 patients were included in the analysis. A total of 5 imaged regions (bilateral feet/ankles [n = 3], bilateral hands/wrists [n = 2]) from these 4 patients were analyzed. All 4 patients completed 24 wk of pegloticase treatment (12 infusions) and had sustained SU-lowering response during treatment month 3 (weeks 10–14) and month 6 (weeks 20–24). Their demographics, baseline characteristics, immunosuppression regimens, and relevant concomitant medications are summarized in Table 2. All 4 patients received a stable triple immunosuppression regimen consisting of a calcineurin inhibitor or mTOR inhibitor (tacrolimus or cyclosporine), an antimetabolite (mycophenolate or azathioprine), and prednisone.

**TABLE 1.** Screening and pegloticase treatment characteristics of kidney transplant recipients who underwent serial DECT imaging as part of the PROTECT trial

Characteristics	All patients with DECT imaging (n = 8)	Patients included in V_MSU_ analyses (n = 4)
Patient characteristics		
Male, n (%)	8 (100%)	4 (100%)
Age, y, mean ± SD	52.3 ± 11.2	53.0 ± 14.9
Race, n (%)		
Black	5 (62.5%)	1 (25.0%)
White	3 (37.5%)	3 (75.0%)
Body mass index, kg/m^2^, mean ± SD	32.3 ± 8.7	26.3 ± 6.7
Kidney characteristics		
Time from kidney transplant, y, mean ± SD	18.7 ± 6.9	22.1 ± 5.4
eGFR, mL/min/1.73 m^2^, mean ± SD	45.6 ± 12.4	44.4 ± 17.4
UACR, mg/g, median (range)	327 (98–3133)	382.5 (98–581)
Gout characteristics		
Time since gout diagnosis, y, mean ± SD	11.9 ± 17.0	19.9 ± 22.3
SU at screening, mg/dL, mean ± SD	10.4 ± 2.1	10.5 ± 2.3
No. of acute gout flares in prior 6 mo	5.3 ± 4.6	5.0 ± 5.3
Pegloticase treatment		
No. of pegloticase infusions received	9.8 ± 4.2	12.0 ± 0.0
Premature discontinuations due to COVID concerns^*a*^	2 (25.0%)	0
12 infusions received	6 (75.0%)	4 (100%)
Treatment response (SU <6 mg/dL for ≥80% of month)		
Month 3 (weeks 10–14)	6 (75.0%)	4 (100%)
Month 6 (weeks 20–24)	6 (75.0%)	4 (100%)
eGFR at week 24, mL/min/1.73 m^2^, mean ± SD	47.4 ± 7.0	47.4 ± 9.6
UACR at week 24, mg/g, median (range)	506 (20–1988)	386 (20–578)

BMI is based on day 1 values.

^*a*^Two subjects discontinued because of COVID concerns (2 and 4 infusions received).

BMI, body mass index; DECT, dual-energy computed tomography; eGFR, estimated glomerular filtration rate; SU, serum urate; UACR, urine albumin-creatinine ratio; V_MSU,_ monosodium urate volume.

**TABLE 2.** Demographics, baseline characteristics, and immunosuppression regimens in the 4 patients included in the analysis

	Age, y	Race	Time since gout diagnosis, y	eGFR, mL/min/1.73 m^2^	Time since transplant, y	No. of acute gout flares in prior 6 mo	Immunosuppression regimens	Relevant concomitant medications
Patient 1	33	White	5	25.0	7.2	6	Cyclosporine, mycophenolic acid, prednisone	Escitalopram (SSRI), cholecalciferol, calcium carbonate
Patient 2	51	White	18	63.8	27	2	Mycophenolate mofetil, prednisone	Escitalopram (SSRI), cholecalciferol
Patient 3	67	White	51	35.5	17	12	Tacrolimus, mycophenolate mofetil, prednisone	Levothyroxine (thyroid hormone)
Patient 4	61	Black	4	53.3	26	0	Cyclosporine, azathioprine, prednisone	Calcitriol

eGFR, estimated glomerular filtration rate; SSRI, selective serotonin reuptake inhibitor.

### SU Level and Deposited MSU Changes During Pegloticase Therapy

**FIGURE 1.**
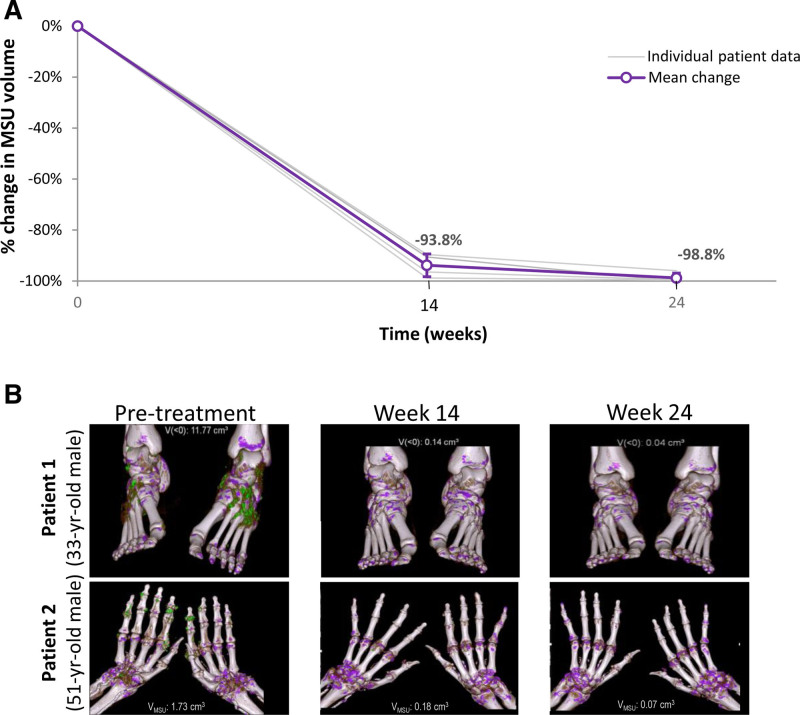
Intense SU-lowering and decrease in V_MSU_ with pegloticase seen in DECT imaging. A, All patients received 24 wk of pegloticase therapy with sustained urate lowering during month 6 of treatment. Gray closed circles represent % reduction in individual scans, and open purple circles show mean % change in V_MSU_ across imaged patients. Error bars represent SD. B, Examples of serial DECT imaging in 2 patients who received 24 wk of pegloticase therapy. Patient 1 (screening SU: 13.4 mg/dL, eGFR: 25.0 mL/min/1.73 m^2^, UACR: 356) had a 5-y history of gout with 6 flares in 6 mo before screening. Patient 2 (screening SU: 7.8 mg/dL, eGFR: 63.8 mL/min/1.73 m^2^, UACR: 581) had an 18-y history of gout with 2 flares in 6 mo before screening. Deposited MSU is shown in green, and individual scan V_MSU_ measurements are shown. DECT, dual-energy computed tomography; eGFR, estimated glomerular filtration rate; SU, serum urate; UACR, urine albumin-creatinine ratio; V_MSU_, monosodium urate volume.

Notably, intense SU-lowering with pegloticase was observed in all 4 patients at weeks 14 and 24 (Table 3). V_MSU_ rapidly and progressively decreased in all imaged regions included in DECT analyses. At week 24 of pegloticase treatment, the mean patient-level change from screening in V_MSU_ was –98.8% ± 1.9% (n = 4 patients; Table 3; Figure 1A). Representative serial DECT imaging in 2 patients is shown in Figure 1B. The mean region-level change from screening was –94.4% ± 4.1% and –98.9% ± 1.7% at weeks 14 and 24, respectively (n = 5 imaged regions).

**TABLE 3.** Pegloticase treatment, SU levels, and serial V_MSU_ in the 4 patients included in the analysis

	Pegloticase treatment		Serum urate, mg/dL			V_MSU_^*a*^ in imaged regions			% Change from screening		Imaged region
	No. of infusions	6-mo responder	Screening	Week 14	Week 24	Screening	Week 14	Week 24	Week 14	Week 24	
Patient 1	12	Yes	13.4	<1.0	<1.0	11.77	0.14	0.04	–98.8%	–99.7%	Feet/ankles
Patient 2	12	Yes	7.8	<0.2	2.8	1.73	0.18	0.07	–89.6%	–96.0%	Hands/wrists
Patient 3	12	Yes	10.5	<0.2	<0.2	0.53	0.05	0.00	–90.6%	–100%	Feet/ankles
Patient 4	12	Yes	10.3	<0.2	<0.2	30.33	1.09	0.13	–96.4%	–99.6%	Feet/ankles, hands/wrists
% or mean ± SD	12	100%	10.5 ± 2.3	NA	NA	11.1 ± 13.8	0.4 ± 0.5	0.1 ± 0.1	–93.8 ± 4.5%	–98.8 ± 1.9%	

^*a*^Total of all imaged regions with screening V_MSU_ ≥0.5 cm^3^.

MSU, monosodium urate;SU, serum urate; V_MSU_, MSU volume.

### Bone-related Imaging Findings

**FIGURE 2.**
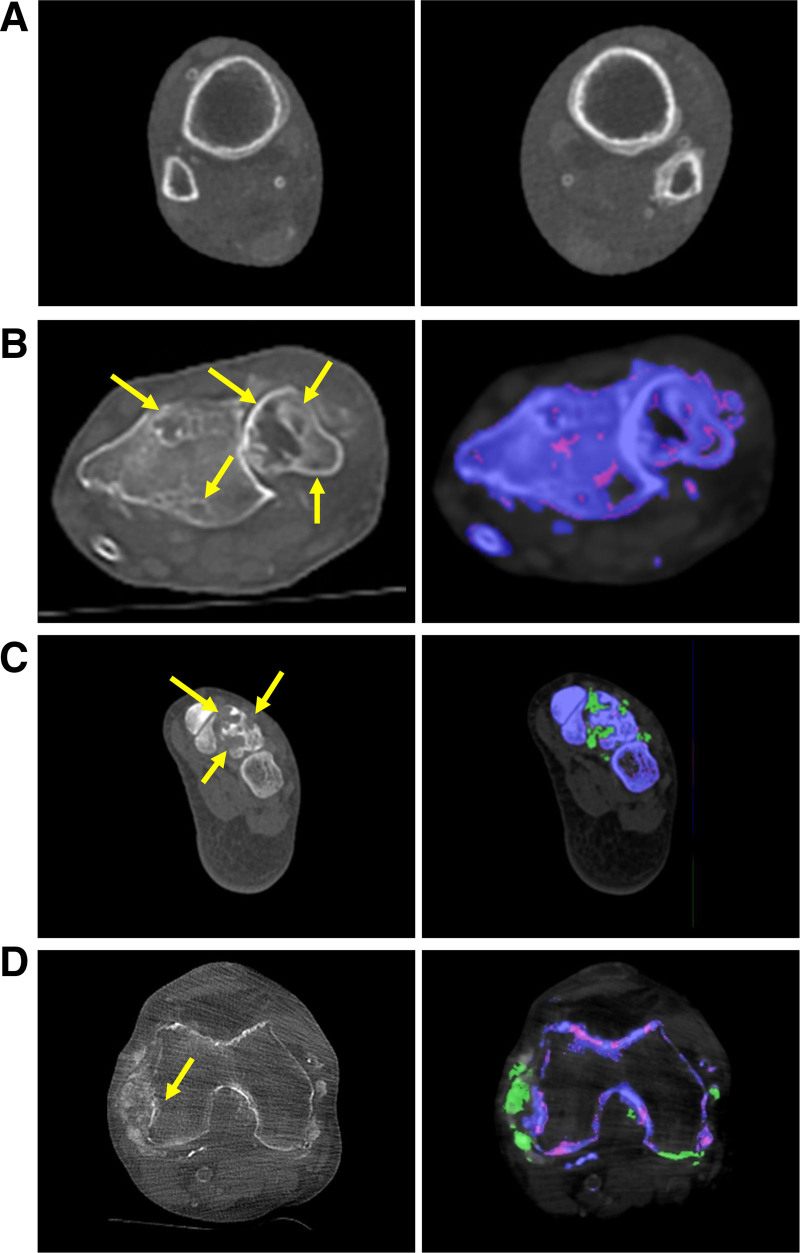
Bone-related DECT findings show osteopenia/osteomalacia (A) and multiple bone erosions (B–D) before pegloticase treatment. A, Axial CT image of the bilateral tibia and fibula before treatment demonstrates subperiosteal and intracortical resorption with diffuse osteopenia and periosteal reaction. B, Axial CT image of the wrist before treatment demonstrates multiple erosive-type lesions within the distal radius and ulna (yellow arrows). There is an amorphous mineralized matrix in the adjacent soft tissues. DECT images do not demonstrate that this mineralized matrix represents MSU deposits. C, Axial CT image of the bilateral feet demonstrates multiple erosions at the bases of the third and fourth metatarsals (arrows). DECT imaging reveals MSU deposits (green) adjacent to the erosions. D, Axial CT image of the knee demonstrates diffuse osteopenia. Amorphous mineralized matrix (white) is adjacent to the medial femoral condyle with adjacent erosion (yellow arrow). DECT imaging demonstrates both MSU deposits (green) and unknown mineralized material (whitish purple) adjacent to the bone erosion. DECT, dual-energy computed tomography; MSU, monosodium urate.

Radiological findings suggested poor overall bone health in this KT recipient population before pegloticase treatment. Images were suggestive of osteopenia/osteomalacia, seen as cortical thinning, subperiosteal/intracortical/endosteal bone resorption, and trabecular bone loss (Figure 2A). Furthermore, bone erosions were common in hands/wrists and feet/ankles, with ≥10% erosion in 2 of 4 patients at screening. Three types of erosions were observed: those adjacent to MSU color-coded deposits, those adjacent to non-MSU color-coded mineralized matrix, and those not adjacent to any material deposits (Figure 2B–D). In some cases, erosions were adjacent to both MSU and non-MSU color-coded deposits.

**FIGURE 3.**
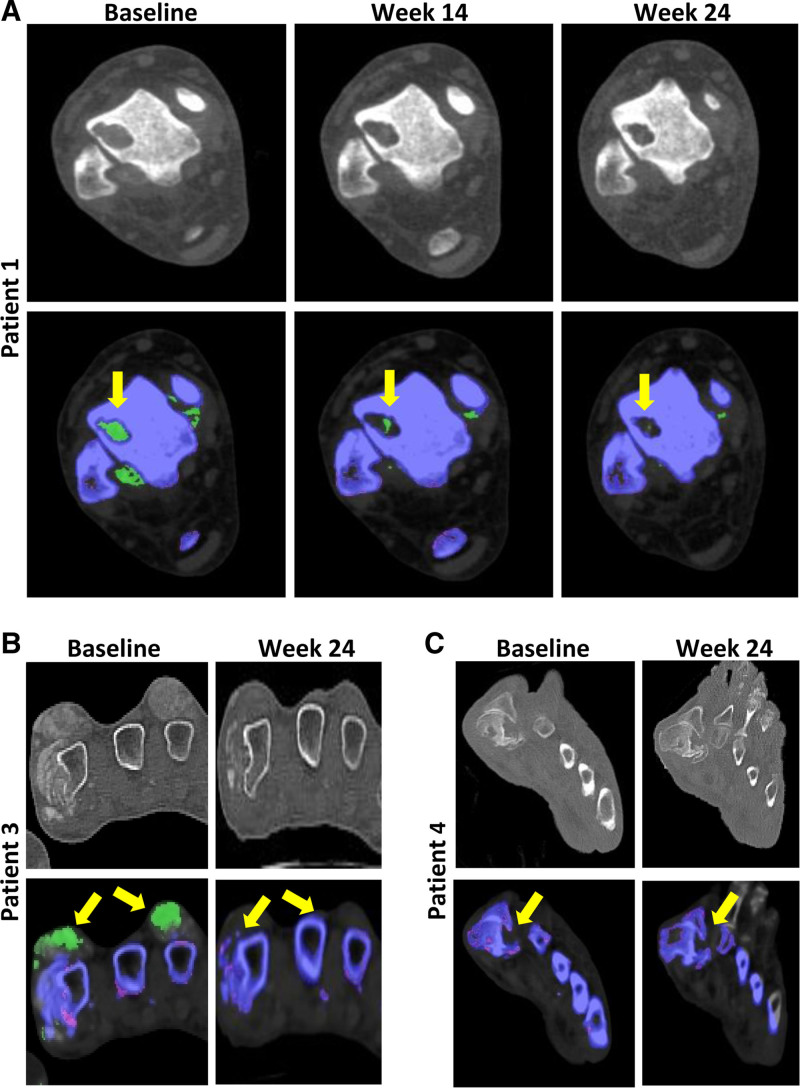
Serial DECT images during pegloticase treatment showing the 3 types of observed erosions: adjacent to urate (A; axial CT, talus of the ankle), adjacent to both color-coded MSU and other mineralized matrix (B; axial CT, metacarpals of hand), and not adjacent to a color-coded MSU deposit (C; long axis CT, first metatarsal). Concomitant with urate deposit depletion, the urate-adjacent erosion had decreased in size by week 24 in this patient without radiologic features of CKD-MBD (A). No changes in the other erosion types were observed in patients with radiologic features of MBD (B and C). Urate deposition is represented in green. Other mineralized deposition is represented in white/purple. CKD-MBD, chronic kidney disease-mineral and bone disorder; CT, computed tomography; DECT, dual-energy CT; MSU, monosodium urate.

Available serial DECT images of bone erosions revealed that most erosions persisted through the 24-wk pegloticase treatment period. These included bone erosions that were MSU-adjacent, non-MSU deposit-adjacent, and deposit-independent erosions (Figure 3B–D). However, the single patient (patient 1) with relatively normal-appearing bone mineralization on CT imaging had an improvement in the size of a large MSU-adjacent bone erosion at weeks 14 and 24 (Figure 3A).

## DISCUSSION

Hyperuricemia and gout are common and often undertreated in KT recipients. When SU levels remain >6 mg/dL, MSU crystals can deposit in joints and surrounding tissues, and if they remain untreated or undertreated, they may lead to a heavy urate burden and subsequent urate-adjacent bone erosions.^15^ In the PROTECT clinical trial, pegloticase successfully lowered SU levels in KT recipients with uncontrolled gout.^12^ In agreement with prior studies in nontransplant patients,^6,9^ previously published PROTECT imaging data have confirmed near depletion of urate deposits after 24 wk of sustained intensive SU-lowering with pegloticase. Importantly, patients also had stable graft function, quality-of-life improvements, and a marked reduction of gout flares by the end of the study.^13^ Together, these findings demonstrate a positive impact of SU management and urate depletion on the overall well-being of KT recipients with uncontrolled gout. However, further study is needed to better understand how effective gout and SU management may be beneficial to longer-term renal outcomes in KT recipients.

In nontransplant populations, MSU crystal deposition has been shown to be proximally associated with bone erosions, with tophus volume positively correlating to erosion volume.^15^ Bone erosion repair has not been commonly seen with oral urate-lowering therapies, but there is a report of the xanthine oxidase inhibitor topiroxostat (up to 120 mg) with benzbromarone leading to bone erosion remodeling after 30 mo of treatment.^16^ However, DECT studies in nontransplant gout patients have shown evidence of remodeling of gout-related bone erosions after aggressive pegloticase treatment for 12 mo.^6,9^ Similar observations were made in the current study, but only in urate-adjacent bone erosions in a single patient without imaging evidence of CKD-MBD. In contrast to transplant-naive patients, erosions that were not adjacent to urate were also observed in the current study, including those adjacent to non-urate mineralized matrix deposits and those not adjacent to any deposited material. These MSU crystal-independent erosions may be related to CKD-MBD and/or mineralization disorders secondary to chronic renal insufficiency and may be independent of gout. Therefore, these deposits and erosive-type bone lesions did not change with intensive SU-lowering. Bone disease in KT recipients is highly complex, and non-urate erosions could have multiple etiologies, which is out of the scope of this study.

Patients with kidney dysfunction are prone to CKD-MBD, particularly KT recipients, due to the severity and often long history of CKD. Hyperuricemia and gout have also been associated with bone abnormalities. The literature examining CKD-MBD in patients with and without gout is sparse. However, hyperuricemia has been associated with secondary hyperparathyroidism and subsequent risk for hypovitaminosis D.^17^ Given that the pathogenic mechanisms are independent of one another, it may be that KT recipients with uncontrolled gout are at particular risk for bone damage. One study showed higher prevalence of CKD-MBD in CKD patients with gout than in their counterparts without gout despite similar demographics and renal characteristics.^18^ Given that KT recipients have a high risk for bone and mineral disorders^10^ and that gout-related damage is both preventable and potentially modifiable with SU control, the potential benefit of effective gout management is an opportunity to improve patient health.

This study had several limitations including its small sample size, lack of a placebo/control group, open-label design, and heterogeneity of immunosuppression regimens among patients. Furthermore, possible CKD-MBD-related laboratory values (ie, parathyroid hormone, phosphorous, alkaline phosphatase, vitamin D) were not measured as part of the PROTECT trial, preventing further information on CKD-MBD from being elucidated. Therefore, further study is needed to better understand the relative contributions of CKD-related and gout-related bone disease. Additionally, other confounders of bone health, including age, medications, length of steroid use, current CKD stage, and parathyroid function, were not examined and may have played a role in the presence/absence of bone radiological improvements. Notably, the PROTECT trial was not designed to examine overall bone health or changes in bone health with intensive SU-lowering. However, our findings raised novel questions about bone health in this population. To our knowledge, bone- and soft tissue-related findings in KT recipients using DECT imaging have not yet been reported.

In conclusion, successful pegloticase therapy for 24 wk resulted in near complete depletion of MSU crystal deposits in these KT recipients. Furthermore, DECT imaging suggested overall bone health in KT recipients with uncontrolled gout was poor, with both gout-related and CKD-related mechanisms likely involved. Interestingly, in the single patient without imaging evidence of osteopenia/osteomalacia, gout-related erosion remodeling was observed with intensive urate-lowering and subsequent MSU depletion. These data provide novel insights into bone health in KT recipients with uncontrolled gout and responses of bone erosions after SU control in this vulnerable population.

## ACKNOWLEDGMENTS

Editorial support for this article was provided by Swati Ghatpande, PhD, an employee of and stockholder in Amgen Inc.
